# What Is the Structure of Time? A Study on Time Perspective in the United States, Poland, and Nigeria

**DOI:** 10.3389/fpsyg.2018.02078

**Published:** 2018-11-01

**Authors:** Małgorzata Sobol-Kwapińska, Tomasz Jankowski, Aneta Przepiorka, Ike Oinyshi, Piotr Sorokowski, Philip Zimbardo

**Affiliations:** ^1^Department of Psychology, University of Wrocław, Wrocław, Poland; ^2^Department of Psychology, The John Paul II Catholic University of Lublin, Lublin, Poland; ^3^Department of Psychology, University of Nigeria, Nsukka, Nigeria; ^4^Department of Psychology, Stanford University, Stanford, CA, United States

**Keywords:** time perspective, factor structure, ZTPI, past, present, future

## Abstract

The aim of this article was to analyze the fit of the model of time perspective, measured by the Zimbardo Time Perspective Inventory (ZTPI; Zimbardo and Boyd, 1999), to data collected in three countries: the United States (*N* = 283), Poland (*N* = 510), and Nigeria (*N* = 357). Confirmatory factor analysis, exploratory structural equation modeling, an expected parameter change and parallel analysis were used. The best-fitted model of time perspective was the one in the United States, and the least fitted model was the one in Nigeria. Possible sources of misspecifications in the model of time perspective were discussed. We also present an analysis of the fit of the four-factor model of time perspective. The four-factor model was very well fitted in the United States and in Poland. Results were discussed in the context of clock time and event time theory.

## Introduction

What is the structure of time? What elements does time consist of? Seconds, hours, days, weeks, years, the past, the present, and the future? Or perhaps events, good and bad moments that pass slowly or quickly? In the context of psychology, it can be said that what time is like depends on how we think about it. And the way we think about time is related to our personality, life experience, and upbringing (Zimbardo and Boyd, 1999, 2008; Stolarski et al., 2014). A factor that has great influence on the formation of attitudes toward time is the culture (see Zimbardo and Boyd, 1999, 2008; Lang and Carnstensen, 2002; Milfont and Gouvela, 2006). People who have come into contact with different cultures mention differences connected with approach to time as one of the main problems they have to confront in the new place (Levine, 1997; Levine and Norenzayan, 1999; Brislin and Kim, 2003). The significance of attitudes toward time is very accurately captured by the popular expression “silent language,” which Edward Hall used to refer to as the attitude toward time in a given culture (Hall, 1990). Therefore, in order to understand the culture of a particular country, it is necessary to explore the way of treating time that is characteristic of that particular place.

One of the aspects of attitudes toward time is the attitude toward clock time. Clock time imposes order on everyday activities and facilitates the functioning of human groups by providing a kind of frame of reference, which determines the times of beginning and ending meetings, events, and other forms of activity. People differ in the degree to which they live by the clock-based order. In cultural terms, there is a distinction between so-called clock-time cultures and event-time cultures (Hall, 1990; Levine, 1997; Avnet and Sellier, 2011). In clock-time cultures, great importance is attached to the clock and to times or deadlines defined in terms of specific hours. By contrast, in event-time cultures the passage of time is defined by events, and people do not attach great weight to times set in advance. A new task or event begins when the previous one has come to an end. People in Western societies organize their time mainly according to the clock. In the case of event time being predominant, a task or event begins when the previous one is over.

The clock determines the basic intervals of the passing time – seconds, minutes, and hours. A person also divides time into larger intervals – namely, into the time that has passed, the time that is going on, and the time that is to come. It is these three basic dimensions of time to which the so-called time perspective is related. Zimbardo and Boyd (1999) understand it – in the most general terms – as a tendency to focus on the past, the present, or the future, combined with a positive or negative evaluation of a particular dimension of time. The time perspective theory postulates that, to some extent unconsciously, a person divides personal experience into the past, the present, and the future. An important consequence of this process is the fact that a particular person’s judgments, decisions, and activities are influenced by the dimension of time this person prefers (see Zimbardo and Boyd, 1999, 2008).

Thus understood, time perspective, measured by the Zimbardo Time Perspective Inventory (ZTPI; Zimbardo and Boyd, 1999), has been the subject of many studies in the United States and in other countries. Their results have revealed that it is an important psychological variable associated with many areas of human functioning, such as well-being, health behaviors, risky behaviors, tendency to become addicted, etc. (e.g., Zimbardo and Boyd, 1999, 2008; Carelli et al., 2011; Zhang and Howell, 2011).

Zimbardo and Boyd (1999) distinguished five types of time perspectives: past-positive perspective – a tendency to focus on the positively evaluated past; past-negative perspective – a tendency to focus on the negatively evaluated past; future perspective – a tendency to think about the future in terms of goals to be achieved and tasks to be done; present-hedonistic perspective – a tendency to take advantage of pleasure “here and now” at all costs; and present-fatalistic perspective – a tendency to passively exist in the present, stemming from the belief that life is governed by fate. This five-element structure of time perspective was obtained in an exploratory principal component factor analysis (with varimax rotation) of the results of a study conducted on a sample of American university students (Zimbardo and Boyd, 1999).

The five-factor structure yielded by exploratory analyses was confirmed in other studies (e.g., Díaz-Morales, 2006). In some studies, exploratory factor analyses suggested a different number of factors. Worrell and Mello (2007) obtained EFA results indicating a possible six-factor solution with negative attitude toward the future as the sixth factor. The study by Sobol-Kwapinska et al. (2016), conducted in Poland and concerning the structure of time perspective measured by the ZTPI in three age groups – aged 18–27, 28–39, and 40–65 – revealed that only in the youngest group was structure of time perspective the same as in the original model of time perspective by Zimbardo and Boyd (1999). The results of analyses using principal axis factor extraction with Promax (oblique) rotation in a group of Poles suggested a four-factor solution, without a factor responsible for the present-fatalistic perspective (Przepiorka et al., 2016).

With regard to confirmatory analyses of the original version of the ZTPI, in different studies the CFI usually did not reach the acceptable level. Worrell and Mello (2007) tested 815 American teenagers (aged 11–18). They obtained the following indices in their confirmatory factor analysis (CFA) of the five-factor model: RMSEA = 0.055 to 0.059, SRMR = 0.057, CFI = 0.636. In the study by Milfont et al. (2008), conducted in Brazil (247 students, mean age: 22.47), the following fit indices were obtained for the five-factor model: RMSEA = 0.078, SRMR = 0.099, CFI = 0.7. The study by Boniwell et al. (2010), conducted in the United Kingdom (179 subjects, aged 18–58), yielded the following fit indices in CFA: RMSEA = 0.045, SRMR = 0.08, CFI = 0.975. Similarly, in a study conducted in Sweden (419 subjects aged 18–80) Carelli et al. (2011) obtained the following fit indices: RMSEA = 0.06, SRMR = 0.09, CFI = 0.63. A confirmatory analysis of results obtained in a study using the Polish version of the ZTPI, conducted on a sample of 1,000 subjects aged 18–78, yielded the following indices: RMSEA = 0.053 (from 0.052 to 0.055), SRMR = 0.08, CFI = 0.79, and TLI = 0.733 (Sobol-Kwapinska et al., 2016). Worrell et al. (2016) conducted research in the United States, Australia, the United Kingdom, and Slovenia. They obtained the following fit indices: RMSEA = 0.048 to 0.058, SRMR = 0.07 to 0.10, CFI = 0.61 to 0.71, and TLI = 0.59 to 0.7. Davis and Ortiz (2017) tested 748 subjects (aged 18–70) in the United States. They obtained the following fit indices for the five-factor model: RMSEA = 0.065 to 0.069, SRMR = 0.089, CFI = 0.879. To sum up, not all the general fit indices of the ZTPI full form reached the level regarded as acceptable, especially CFI was too low.

Several short versions of the ZTPI have been developed (e.g., Zhang et al., 2013; Sircova et al., 2014; Przepiorka et al., 2016). A vast majority of items from particular scales were eliminated on the basis of statistical analyses. However, these short versions were usually not confirmed in follow-up studies conducted by other researchers (see Mckay et al., 2014; Worrell et al., 2016). Recently, Worrell et al. (2016) published a study in which they presented a short version of the ZTPI, but the items were eliminated by means of a theoretical method – the authors removed those items that did not contain time words. The version proposed by Worrell et al. (2016) was found to have good fit indices in the United States, Australia, the United Kingdom, and Slovenia, however, the reliability coefficients of scales were mostly below the acceptability level.

The aim of the presented research is to analyze the fit of the model of time perspective according to Zimbardo and Boyd (1999) to data collected in three countries: the United States, Poland, and Nigeria. These countries were selected in such a way as to represent cultures with strong (United States), moderate (Poland), and weak (Nigeria) tendencies toward clock-based structuring of time.

In the United States, people live according to clock time, under pressure of time (Tarkowska, 1992; White et al., 2011). Time is a resource that is necessary to use in such a way as to achieve a high social status and increasingly good results at work (Chua and Rubenfeld, 2014). The results obtained by Levine et al. (1980) reveal the great importance that Americans attach to punctuality. Poles exhibit a moderate tendency to plan and structure time. Poland is a country in which time functions as a principle organizing private and social life to a moderate degree (e.g., Tarkowska, 1992; Nosal and Bajcar, 2004). Nigerians are a society dependent to a very small degree on quantified clock time. In the literature, there are references to “African time,” an expression describing the belief that one should not hurry anywhere because everything may just as well be done later (Hall, 1990; Opata, 2002; Dissel and Potgieter, 2007).

We expected that the weaker the clock-based structuring of time, the less well fitted to the data the model of time perspective according to Zimbardo and Boyd (1999) would be. This hypothesis was based on the model of temporal relations. According to Nosal and Bajcar (2004), what underlies the experience of time are mental representations of time – that is, various conceptualizations of time. Conceptualizations of time as measurable and divisible are associated with a tendency to segment time, to divide it with clear borders defined in advance. In the case of a weak tendency to conceptualize time as a measurable and divisible resource, the division of time into predefined segments is also less clear and less important; this also refers to the past–present–future division. That is why we supposed that the weaker clock-based structuring of time should be associated with fewer number of factors in the time perspective structure.

Another aim of our study is to check – in the case of the absence of a confirmation of the original Zimbardo and Boyd (1999) model – possible sources of non-fit to the data. Numerous examples of research indicate a rather high probability of problems that can be encountered in the factor validation of the ZTPI. Our aim, then, is to suggest some changes that may contribute to the future revision of the model, as well as a method based on it for studying basic forms of time perspective.

## Materials and Methods

### Participants

There were 283 American adults (181 female and 102 male; aged 16–72, mean age 23.95; standard deviation 9.78). Most of the respondents had secondary school education (49.3%); others university (28.2%) or primary school education (22.5%). The participants represented followed about 25 different occupations. Most of them worked as blue-collar workers, social workers, and medical professionals. The participants lived in villages (10.5%), in small towns (15.1%), in medium- sized towns (23.5%), in large towns (26.1%), and in big cities (24.8%). As regards the relationship status, 50.5% of the participants were married, 10.4% were single, and 39.1% were in open relationships.

The Polish group consisted of 510 subjects (285 female and 225 male; aged 14–77, mean age 30.62; standard deviation 13.37). Most of the respondents had secondary school education (51.4%); others university (27.1%) or primary school education (21.5%). The participants represented followed about 20 different occupations. Most of them worked as technicians, teachers, blue-collar workers, and social workers. The participants lived in villages (12.4%), in small towns (30.1%), in medium- sized town (26.7%), in large towns (28%), and in big cities (2.8%). As regards the relationship status, 58.5% of the participants were married, 10.4% were single, and 31.1% were in open relationships.

There were 357 Nigerian adults (176 female and 181 male; aged 16–59, mean age 25.46; standard deviation 6.5). Most of the respondents had secondary school education (67%); others university (21.3%) or primary school education (11.7%). The participants represented about 15 different occupations. Most of them worked as social workers, teachers, farmers, and medical professionals. The participants lived in villages (32.3%), small towns (30.1%), medium- sized town (25,1%), large towns (10.1%), and big cities (2.4%). As regards the relationship status, 50.5% of the participants were married, 10.4% were single, and 39.1% were in open relationships.

The participants were recruited from the general population, through social networking websites and also through a university course. The respondents were selected at random. The participants received no remuneration for taking part in the study. Participation in the research was voluntary. When the participants consented to the research, they completed paper questionnaires received from researchers or research assistants. The research was conducted in accordance with ethical standards. The consent procedure and study protocol received ethical approval from the Catholic University of Lublin Institute Ethics Committee. The participants received a request to take part in the study and they were informed about the aim of the study and about the confidentiality of their answers. All subjects gave written informed consent in accordance with the Declaration of Helsinki.

### Measures

#### Time Perspective

The time perspective was measured by Zimbardo and Boyd’s (1999) ZTPI. In the United States and Nigeria, the original version of ZTPI was used; in Poland we used the Polish adaptation of ZTPI (Przepiorka et al., 2016). The ZTPI consists of five scales (56 items). The Past-Positive scale (9 items, e.g., *“Happy memories of good times spring readily to mind.”*) assesses focusing on a positive assessment of the past. The Past-Negative (10 items, e.g., *“I think about the bad things that have happened to me in the past.”*) assesses the tendency to concentrate on the negative past. The Present-Hedonistic scale (15 items, e.g., *“I make decisions on the spur of the moment.”*) focuses on current pleasures without considering the consequences of one’s behavior. The Present-Fatalistic scale (9 items, e.g., *“Fate determines much in my life.”*) focuses on the present associated with the belief that the future is out of control. The Future scale (13 items, e.g., *“I complete projects on time by making steady progress.”*) assesses focusing on planning and formulation of goals. The respondent answers using a five-point Likert scale (from 1 – *very uncharacteristic* to 5 – *very characteristic*).

### Procedure and Statistical Analyses

We calculated the percentage of missing items per instrument by dividing the number of the missing items by the total number of the items to be scored. The records with more than 20% of the items missing were excluded. In the case of records with up to 20% missing items, the scores were prorated by introducing the average of the non-missing items. To analyze the structure of time perspective measured by the ZTPI, we used CFA. We employed maximum likelihood (ML) estimation, which is applicable for normally distributed data and continuous variables. With regard to the ways of determining the model fit in CFA, researchers are not unanimous on this issue. A very popular practice among scholars is the use of general goodness-of-fit (GoF) indices, mainly the comparative fit index (CFI), root mean square error of approximation (RMSEA), and standardized root mean square residual (SRMR) (Kline, 2011). Researchers usually adopt the GoF acceptability criteria proposed by Hu and Bentler (1999, pp. 26–27): >0.95 for CFI, <0.06 (*N* ≥ 250) and <0.08 (*N* < 250) for RMSEA, and <0.11 for SRMR. Some researchers claim that there is no rational support for adopting fixed cut-off criteria for GoF (see Marsh et al., 2004; Fan and Sivo, 2005; Barrett, 2006; Saris et al., 2009). Some believe that these cut-offs are too restrictive, particularly for questionnaires consisting of a large number of items (Saris et al., 2009; Perry et al., 2013; Ropovik, 2015). Perry et al. (2013) point out that in social sciences, factor loadings are usually lower, and therefore excessively restrictive cut-off criteria frequently lead to erroneous results. In social sciences, a model very well fitted to the data is very rare, which stems from the specificity of these sciences (see Saris et al., 2009; Ropovik, 2015; Greiff and Heene, 2017). Moreover, Saris et al. (2009) claim that general GoF indices vary to a very large extent, depending on the values of parameters that are incidental and unrelated to model misspecification. Greiff and Heene (2017) also stress the undeniable fact that GoF is strongly affected by factors unrelated to model fit.

It is not possible to determine universal cut-off criteria, independent of the nature of a particular model (see Beauducel and Wittmann, 2005; Sharma et al., 2005; Yuan et al., 2005; Ropovik, 2015; Greiff and Heene, 2017). Relying exclusively on the values given by Hu and Bentler (1999) may therefore lead to the approval of a model containing misspecifications and to the rejection of a well-fitted model (Greiff and Heene, 2017). Saris et al. (2009) mention the fact that on the basis of general fit indices, it is impossible to determine the “size” of model misspecification. Hu and Bentler (1999) themselves warned against treating their cut-off criteria as golden rules.

Some scholars believe that the best model fit index is χ^2^ and that only this index should be used (see Ropovik, 2015; Greiff and Heene, 2017). Greiff and Heene (2017) point out, however, that the interpretation of χ^2^ involves problems similar to those involved in GoF. The value of χ^2^ is influenced by the values of factor loadings and by sample size.

The best currently suggested solution is the careful analysis of detailed model fit (Chen et al., 2001; McIntosh, 2007; Ropovik, 2015; Greiff and Heene, 2017). However, there is no established, universal procedure for this kind of analysis. As an alternative method of determining model correctness, Saris et al. (2009) propose the use of expected parameter change (EPC) combined with modification index (MI) and the power of the MI test. We used this method proposed by Saris et al. (2009). We also used exploratory structural equation modeling (ESEM; Asparouhov and Múthen, 2009) to analyze the structure of time perspective. ESEM is a statistical analysis that integrates confirmatory and exploratory factor analyses. It can be said that ESEM is less restrictive than CFA because ESEM does not constrain the non-target loadings to be zero. The number of factors in the model is specified in ESEM. ESEM yields typical CFA parameters. Due to the properties of this analysis, many scholars encourage the use of ESEM (see Asparouhov and Múthen, 2009; Marsh et al., 2011; Worrell et al., 2016).

To sum up, in our study, we analyzed the fit of the model of time perspective to the data using a procedure that comprised the following stages: (1) measurement of the model’s general GoF using CFA and ESEM; and (2) measurement of the model’s specific GoF by an application of the method designed by Saris et al. (2009), analysis of factor loadings and factor correlations, analysis of cross loadings, analysis of missing paths, and error correlations.

The above analyses were performed separately for scores from the United States, Poland, and Nigeria. We used the lavaan, semTools, paran and GPA rotation packages in R program (R Development Core Team, 2011).

## Results

R software (R Development Core Team, 2011) was used for data analysis. First, we tested the general fit of the original five-factor model of time perspective. Next, we computed a detailed analysis of the fit of this model: the factor loadings, cross-loadings, factor correlation, and the expected change parameters change were calculated. Subsequently, the parallel analysis method was applied to determine the number of factors of ZTPI. Later, the confirmatory analyzes of the four-factor model of time perspective were conducted.

We calculated the percentage of missing items per instrument by dividing the number of the missing items by the total number of the items to be scored. The records with more than 20% of the items missing were excluded. In the case of records with up to 20% missing items, the scores were prorated by introducing the average of the non-missing items.

### Descriptive Statistics

Descriptive statistics and internal consistency for the ZTPI scales in the United States, Poland, and Nigeria are presented in Table 1. We computed Cronbach’s alpha as well as omega coefficients, using the psych package (Revelle, 2018). The reliability coefficients of ZTPI scales in the United States and in Poland are acceptable and comparable with the values obtained by other researchers. In the United States, the scales have the highest reliability; in Nigeria, the Past-Positive and Present-Hedonistic scales had very low reliability coefficients – particularly the Past-Positive scale.

**Table 1:** Psychometric properties of ZTPI scales in the United States, Poland, and Nigeria.

Country	Scale	*M*	*SD*	Cronbach’s alpha	Omega	coefficients
United States	Past-positive	31.78	6.14	0.80	0.80
	Past-negative	30.45	7.09	0.82	0.82
	Present-fatalistic	23.26	5.54	0.71	0.67
	Present-hedonistic	48.88	7.93	0.78	0.80
	Future	46.88	6.76	0.73	0.77
Poland	Past-positive	32.87	32.87	0.64	0.65
	Past-negative	30.11	30.11	0.82	0.83
	Present-fatalistic	24.31	24.31	0.68	0.68
	Present-hedonistic	51.55	51.55	0.74	0.75
	Future	46.08	46.08	0.78	0.78
Nigeria	Past-positive	30.46	4.11	0.20	0.12
	Past-negative	34.09	6.81	0.74	0.73
	Present-fatalistic	23.86	6.25	0.66	0.65
	Present-hedonistic	50.21	7.09	0.56	0.49
	Future	49.03	7.02	0.68	0.67

### The General Fit of the Model of Time Perspective

Firstly, χ^2^ and then GoF (CFI, RMSEA, and SRMR) were calculated (see Table 2). For all models in all three countries, χ^2^ was statistically significant. The CFI index was the highest in the sample of Americans and the lowest in the sample of Nigerians. In ESEM, CFI in the group of Nigerians was lower than in the remaining two groups. The value of RMSEA was comparable in CFA performed in the three countries, and in ESEM it was the highest in the Nigerian group. In CFA, the SRMR index was the highest in the group of Americans, and in ESEM it was highest in the group of Poles. The values of χ^2^ and GoF suggest the best fit of the original five-factor model to data in the group of Americans and the lowest fit in the group of Nigerians.

**Table 2:** Goodness-of-Fit measures for ZTPI scores.

Model		χ2			*df*			χ2/*df*			CFI			RMSEA			SRMR		
		United States	Poland	Nigeria	United States	Poland	Nigeria	United States	Poland	Nigeria	United States	Poland	Nigeria	United States	Poland	Nigeria	United States	Poland	Nigeria
Five factors ZTPI	CFA	3494.70	4413.61	4204.67	1475	1476.12	1475.32	2.37	2.99	2.85	0.59	0.59	0.38	0.07	0.06	0.07	0.10	0.09	0.09
	ESEM	2256.59	2571.62	3068.25	1268	1273,08	1267.87	1.78	2.02	2.42	0.80	0.82	0.59	0.05	0.04	0.06	0.05	0.04	0.05
Five factors ZTPI (with correlated errors)	CFA	3339.11	4203.24	4177.64	1471	1468.66	1471	2.27	2.86	2.84	0.62	0.62	0.39	0.07	0.06	0.07	0.10	0.09	–
Four factors PS-ZTPI	CFA	381.83	490.81	514.36	164	164,15	163.81	2.33	2.99	3.14	0.83	0.82	0.55	0.07	0.07	0.08	0.07	0.07	0.08
	ESEM	211.59	343.06	295.77	116	115.90	115.99	1.82	2.96	2.55	0.93	0.87	0.77	0.06	0.07	0.07	0.04	0.04	0.05
Four factors PS-ZTPI (with correlated errors)	CFA	299.36	353.22	488.05	157	156.54	157.54	1.91	2.25	3.10	0.90	0.91	0.59	0.06	0.05	0.08	0.06	0.05	0.07

*CFI, comparative fit index; RMSEA, root mean square error of approximation; SRMR, standardized root mean square residual; χ2 = Chi-square test; df = degrees of freedom.*

### Detailed Analysis of the Fit of the Model of Time Perspective

In view of the ambiguity involved in the interpretation of global fit indices, we performed a detailed assessment of the models in different groups; our point of departure was the analysis of factor loadings, cross-loadings, factor correlations, and modification indices for each parameter in a given model (see Saris et al., 2009; Greiff and Heene, 2017).

#### Factor Loadings and Cross-Loadings

The table in Appendix 1 presents standardized item loadings for the ESEM in the United States, Poland, and Nigeria. For the sake of simplicity, we discuss only the results obtained in the better fitted ESEM model. As recommended by Saris et al. (2009) we analyzed how many items in each factor had loadings ≥0.40 and how many items of a given factor had loadings ≥0.40 on other factors. The aggregate results of these analyses are presented in Table 3. The results in the United States and in Poland are fairly similar, except for the Past-Positive scale, in which significantly fewer items had factor loadings ≥0.40 in Poland. Item 25 (“*The past has too many unpleasant memories that I prefer not to think about*”) from the Past-Positive scale had a loading ≥0.40 in all three countries on the factor corresponding to the past-negative time perspective. It is a reverse-coded item whose content refers to negative memories. Moreover, in the United States and in Poland, item 24 (“*I take each day as it is rather than try to plan it out*”) of the Future scale has loadings ≥0.40 on the Present-Hedonistic scale. The situation is similar to the case of the previous item: this is also a reverse-coded item and its content concerns making use of life in the present – that is, the hedonistic perspective. In the United States, item 56 (“*There will always be time to catch up on my work*”) from the Future scale is also reverse-coded and has contents referring to fatalism. In the group of Nigerians, three items of the Present-Hedonistic scale have loadings ≥0.40 on other factors.

**Table 3:** Collective results of the analysis of factor loadings and cross-loadings in the United States, Poland, and Nigeria.

Scale	United States		Poland		Nigeria	
	Items with factor loading ≥0.40 on a given factor	Items with factor loading ≥0.40 on other factors	Items with factor loading ≥0.40 on a given factor	Items with factor loading ≥0.40 on other factors	Items with factor loading ≥0.40 on a given factor	Items with factor loading ≥0.40 on other factors
Past-positive (9 items)	9	1 (item 25, past-negative)	5	1 (item 25, past-negative)	1	1 (item 25, past-negative)
Past-negative (10 items)	8	0	8	0	4	0
Present-fatalistic (9 items)	4	0	4	0	4	1 (item 3, past-negative)
Present-hedonistic (15 items)	5	0	5	0	1	3 (item 28, present-fatalistic; item 26, item 55, future)
Future (13 items)	8	2 (item 56, present-fatalistic; item 24, present-hedonistic)	8	1 (item 24, present-hedonistic)	5	0

To sum up, the analyses of factor loadings and cross-loadings show that the number of items with high loadings on their factors was the highest in the American group and the lowest in the Nigerian group. Likewise, the number of items with cross-loadings was the highest in the group of Nigerians.

#### Factor Correlation

Table 4 presents factor correlations in CFA and in ESEM in the United States, Poland, and Nigeria. In CFA, factor intercorrelations in the Nigerian group turned out to be very high. For instance, the correlation between Past-Positive and Present-Hedonistic was 0.97, which may mean that these two time orientations constitute one dimension in this group of subjects. The high correlation between Past-Positive and Past-Negative (0.71) may also indicate that both orientations represent a similar category of time perception in Nigeria.

**Table 4:** Factor correlations in CFA in the United States, Poland, and Nigeria samples.

Country		Past-positive	Past-negative	Present-fatalistic	Present-hedonistic
United States	Past-positive	–			
	Past-negative	−0.55	–		
	Present-fatalistic	−0.34	0.30	–	
	Present-hedonistic	0.25	0.13	0.16	–
	Future	0.27	0.07	−0.59	−0.10
Poland	Past-positive	–			
	Past-negative	−0.48	–		
	Present-fatalistic	−0.15	0.46	–	
	Present-hedonistic	0.16	0.10	0.55	–
	Future	0.32	−0.02	−0.34	−0.46
Nigeria	Past-positive	–			
	Past-negative	0.71	–		
	Present-fatalistic	0.23	0.38	–	
	Present-hedonistic	0.97	0.46	0.26	–
	Future	0.70	0.08	−0.43	0.65

#### The Expected Parameter Change

To perform a detailed analysis of the fit of the model of time perspective we applied the method proposed by Saris et al. (2009; see Greiff and Heene, 2017) – EPC estimation based on MIs and the power of the MI test (see Saris et al., 2009). The EPC estimates the size of the misspecification in the case of all fixed parameters. The MI is a significance test (with 1 df) for the misspecification. The power of the MI test and the standard error of the EPC (see Saris et al., 2009) are also taken into account in this method.

Results of the EPC estimation in the United States, Poland, and Nigeria are available from the corresponding author upon request. Using the criteria proposed by Saris et al. (2009), we observed 21 MIs that suggest misspecification of the CFA model in the group of Americans. Five of them refer to cross-loadings and 16 to covariances between error terms. In the group of Poles, we observed a very similar number of MIs (23). However, in Poland eight MIs refer to cross-loadings and 15 to covariances between errors. In Nigeria, there were 30 MIs. Ten MIs indicate significant cross-loadings and 20 refer to covariances between errors. The results show that the number of misspecifications in the model is the highest in the group of Nigerians and the lowest in the group of Americans.

In the next step, we wanted to check whether general model fit improved after permitting correlated errors between items. In United States, Poland and Nigeria errors of items 31 and 42, 4 and 54, 23 and 42, 8 and 23, 4 and 2, 8 and 31, 31 and 44 were correlated. Despite the combination of measurement errors, not all values of indices were acceptable (see Table 2).

### Parallel Analysis of the Structure of Time Perspective

We applied Horn’s (1965) parallel analysis method to determine the number of factors of ZTPI to be retained in the principal component factor analysis. The package paran (Dinno, 2012) running under R Environment was used to perform the calculations. Factors with corrected eigenvalues above 1.00 are considered to be retained. The number of random data sets in the analysis was 500. Scree plots were provided as well. Six factors were suggested to be retained in the analysis in the American group, 11 factors in the Polish group, and 8 factors in the Nigerian group. This may suggest that items of the original version have more than five theoretical constructs.

### Confirmatory Analysis of the Four-Factor Model of Time Perspective

The above analyses of global and specific fit of the original five-factor model of time perspective according to Zimbardo and Boyd (1999) indicate that the lack of fit stems not only from measurement errors common to several items but mainly from the fact that the scales are heterogeneous and that the items measure more than five theoretical constructs. This has been highlighted by other scholars as well (see Worrell et al., 2016). Moreover, specific fit analyses show that another source of misspecifications in all three tested countries is reverse-coded items. Therefore, the next step was to test the fit of the four-factor model of the short version of the ZTPI – PS-ZTPI, which we had developed in our previous study (see Przepiorka et al., 2016). We decided to test this particular model because it is a short version – the scales contain only items with the highest loadings on the respective factors.

PS-ZTPI items were selected based on the results of principal axis factor extraction performed on ZTPI scores. The PS-ZTPI consists of four scales, with five items in each: Past-Negative (items 4, 34, 36, 50, 54), Present-Hedonistic (items 8, 23, 31, 42, 44), Future (10, 13, 21, 40, 45), and Past-Positive (2, 7, 11, 20, 49) (Przepiorka et al., 2016). General fit indices of the short four-factor model obtained in CFA and in ESEM in the American, Polish, and Nigerian groups are presented in Table 2. Compared to CFA results obtained for the five-factor model, general fit indices improved. In the group of Nigerians, however, they still considerably diverge from the values regarded as acceptable. We also tested the specific fit of the model on the basis of EPC estimation, as recommended by Saris et al. (2009). After combining the errors, we computed global model fit indices (see Table 2). Both in the American group and in the Polish group we obtained good overall fit indices.

## Discussion

The aim of this paper was to analyze the fit of the model of time perspective according to Zimbardo and Boyd (1999) to data from three countries: the United States, Poland, and Nigeria. We expected that the model of time perspective according to Zimbardo and Boyd (1999) would be better fitted to the data in the United States than in Poland and Nigeria and that it would be better fitted in Poland than in Nigeria. We used general model fit indices as well as analyzed the specific fit of the model to data using the methods recommended by Saris et al. (2009) and by Greiff and Heene (2017). The results of the analysis of general as well as specific model fit confirmed our hypothesis – the best-fitted model of time perspective was the one in the United States, and the least fitted model was the one in Nigeria.

Although in the United States the original model was the best-fitted, the results suggest that the five-factor structure of the full form of the ZTPI was not supported. Based on a detailed fit analysis, we observed the following possible sources of misspecifications in the five-factor model: (1) items with high cross-loadings; (2) reverse-coded items (items 24, 25); (3) factor intercorrelations; and (4) heterogeneity of scales. These issues should be taken into account when preparing a revised full version of the ZTPI.

Next we have presented an analysis of the fit of the four-factor model of time perspective that is the short version of the ZTPI – PS-ZTPI (Przepiorka et al., 2016). The theory behind the four-factor model was based on the conception of two main dimensions of time perspective: (1) a focus of thoughts, feelings, and behaviors on a particular dimension of time, and (2) positive or negative evaluation of a given dimension (area) of time (see Nosal and Bajcar, 2004). In terms of these two main dimensions, the basic types of time perspective can be distinguished: focus on the positively evaluated past, focus on the negatively evaluated past, focus on the positively evaluated present, focus on the negatively evaluated present, focus on the positively evaluated future, and focus on the negatively evaluated future. Four of the original ZTPI scales correspond to these basic types of time perspective. The fatalistic perspective has a somewhat different nature. It is associated with ambivalent evaluation of the present – if a person believes that the force governing people’s lives is good (for example, that it is a divine being who cares for humans), he or she will tend to evaluate the present positively. It is not positive or negative evaluation of the present that determines the nature of this type of time perspective but the controllability vs. uncontrollability of time and, generally, of life (see Przepiorka et al., 2016). The four-factor model was very well fitted in the United States and in Poland. In Nigeria, by contrast, neither the original five-factor model nor the short, four-factor model are well fitted to the data. The highly interesting results show the specificity of the experience of time in Nigeria – an event-time country. The issue of Nigerians’ time perspective is all the more worth addressing in further studies as there is a scarcity of literature on the conceptualisation of time in Nigeria. It is generally believed that Nigerians, and probably Africans, tend to think of time in terms of whole and as less organized than seen in the Western cultures. Although events in Nigeria are currently scheduled with the clock-based structure, people do not strictly think of time along this structure. People commonly think of time in terms of morning, afternoon, evening, and night (Opata, 2002).

In sum, the results of our analyses may be helpful in developing a revised version of the ZTPI. As regards Nigeria, it seems necessary to introduce much greater changes in the ZTPI to adapt it for subjects from event-time cultures. As far as Americans and Poles are concerned, the results of specific model fit analyses suggest that, in work on the revised ZTPI, the following issues should be taken into account:

1. Removing items with high cross-loadings or replacing them with other items, associated to a greater extent only with the contents of a given scale – the heterogeneity of ZTPI items has been pointed out also by scholars such as Crockett et al. (2009), Worrell et al. (2013), or Worrell et al. (2016). It is also worth mentioning what has been pointed out by Davis and Ortiz (2017), as far as the Past-Positive scale is concerned – namely, that some of the items have negative connotations. Perhaps this stems from the fact that recollecting pleasant situations from the past may evoke regret that the time has passed and a longing to return to it.
2. Removing or reformulating reverse-coded items, particularly items 24 and 25 (see Herrmann and Pfister, 2013).
3. Removing the Present-Fatalistic scale as mixed – namely, as referring to both negative and positive evaluations of a particular dimension of time and as referring to both the present and the future (as well as strongly related to the past in the Nigerian study).
4. Expanding the ZTPI by adding more scales measuring the tendency to focus on the negatively evaluated future (see Carelli et al., 2011) and a scale measuring the tendency to focus on the positively evaluated present.

As regards the weak points of the presented study, one of them is the size of the tested groups, and the low case-to-free parameter ratio can lead to reduced statistical precision. It is also worth to note, that the Nigeria sample was administered the US English-based version of the ZTPI. As English is not the first language for most of Nigerians, this may have had some impact on the understanding of this questionnaire, introducing biases in the participants’ responses, and thus possibly explaining the lack of fit of the tested time perspective models in this sample. Moreover, we did not use research methods other than questionnaires. In further studies, different methods should be used to measure time perspective and the experience of time in general, such as observation diaries and interviews.

It should be stressed that the ZTPI has good criterion validity. In very numerous studies, ZTPI scores were good predictors of various kinds of behaviors and tendencies (e.g., Zimbardo and Boyd, 1999, 2008; Zimbardo et al., 2012). It should also be noted that a similar problem with the statistical fit of the model to data occurs in the case of questionnaires measuring the Big Five (see Herrmann and Pfister, 2013). CFA results for questionnaires measuring the Big Five are discouraging if based on the global fit index (see Hopwood and Donnellan, 2010; Herrmann and Pfister, 2013). According to Pan et al. (2017), researchers using CFA are frequently faced with a dilemma when a model that is well supported by theory does not fit the data in CFA. Herrmann and Pfister (2013) strongly stressed that a questionnaire should not be rejected and its construct validity should not be questioned only on the basis of low GoF indices obtained in CFA. In further work on the revised version of the ZTPI, it is advisable to take this opinion into account and to consider the specific changes in the questionnaire suggested by the results of analyses presented in this article.

## Author Contributions

All authors conceived and designed the work; acquired, analyzed, and interpreted the data for the work; drafted the work and revised it critically for important intellectual content; approved the final version of the manuscript to be published; and agreed to be accountable for all aspects of the work in ensuring that questions related to the accuracy or integrity of any part of the work are appropriately investigated and resolved. MS-K was responsible for the Introduction, Materials and Methods, Results (part of statistics), and Discussion sections. TJ was responsible for the Introduction and Results sections. AP was responsible for the Introduction section. IO was responsible for the Discussion section. PS was responsible for the Introduction section. PZ was responsible for the Materials and Methods section.

## Conflict of Interest Statement

The authors declare that the research was conducted in the absence of any commercial or financial relationships that could be construed as a potential conflict of interest.
