# Supplementary material for: What Is the Structure of Time? A Study on Time Perspective in the United States, Poland, and Nigeria
Source: Front Psychol. 2018 Nov 1;9:2078. doi: 10.3389/fpsyg.2018.02078 (PMC6221929; doi:10.3389/fpsyg.2018.02078)
Supplement: Supplementary file 1 [file Data_Sheet_1.PDF]

	ZTPI1	ZTPI2	ZTPI3	ZTPI4	ZTPI5	ZTPI6
ZTPI1		0.330***	0.011	-0.071*	0.112***	-0.197***
ZTPI2	0.330***		0.041	0.093**	0.040	-0.032
ZTPI3	0.011	0.041		0.228***	0.154***	0.186***
ZTPI4	-0.071*	0.093**	0.228***		0.194***	0.180***
ZTPI5	0.112***	0.040	0.154***	0.194***		0.089**
ZTPI6	-0.197***	-0.032	0.186***	0.180***	0.089**	
ZTPI7	0.075*	0.145***	0.022	-0.021	0.077**	0.064*
ZTPI8	0.098***	-0.004	0.028	0.031	0.085**	-0.169***
ZTPI9	-0.082**	0.116***	-0.022	0.082**	-0.042	0.172***
ZTPI10	0.049	0.181***	0.139***	0.098***	-0.031	0.313***
ZTPI11	0.091**	0.214***	0.051	-0.055	-0.023	0.043
ZTPI12	0.143***	0.054	0.072*	0.094**	0.016	-0.063*
ZTPI13	-0.008	0.133***	0.041	0.048	-0.033	0.206***
ZTPI14	0.036	-0.113***	0.175***	0.101***	0.146***	-0.061*
ZTPI15	0.084**	0.194***	0.135***	0.122***	0.064*	0.129***
ZTPI16	0.081**	0.081**	0.091**	0.268***	0.172***	0.016
ZTPI17	0.146***	0.180***	0.032	-0.064*	-0.005	-0.005
ZTPI18	0.106***	0.206***	0.127***	0.092**	0.009	0.151***
ZTPI19	0.170***	0.201***	-0.026	-0.037	-0.012	-0.015
ZTPI20	0.124***	0.358***	0.056	0.070*	0.046	0.039
ZTPI21	0.015	0.125***	0.076**	0.015	-0.022	0.164***
ZTPI22	-0.086**	-0.024	0.059*	0.151***	0.032	0.076**
ZTPI23	-0.013	0.026	0.172***	0.026	0.030	-0.009
ZTPI24	-0.029	0.077**	-0.111***	-0.025	-0.082**	0.267***
ZTPI25	0.083**	0.115***	-0.190***	-0.231***	-0.111***	-0.098***
ZTPI26	0.198***	0.247***	0.105***	0.043	-0.024	0.027
ZTPI27	-0.082**	-0.011	0.170***	0.346***	0.105***	0.176***
ZTPI28	0.143***	0.059*	0.047	0.025	0.057	-0.072*
ZTPI29	0.114***	0.213***	0.077**	0.073*	0.046	-0.050
ZTPI30	0.041	0.094**	0.052	0.032	-0.010	0.254***
ZTPI31	0.107***	0.032	0.101***	0.028	-0.038	-0.006
ZTPI32	0.044	0.052	0.084**	-0.019	-0.014	-0.133***
ZTPI33	-0.070*	-0.054	0.151***	0.197***	0.133***	0.008
ZTPI34	-0.036	-0.058*	0.189***	0.270***	0.107***	0.106***
ZTPI35	-0.008	-0.010	0.265***	0.164***	0.149***	0.089**
ZTPI36	-0.098***	-0.047	0.194***	0.277***	0.126***	0.197***
ZTPI37	0.142***	0.050	0.024	0.094**	0.063*	-0.225***
ZTPI38	0.007	0.005	0.191***	0.088**	0.156***	-0.035
ZTPI39	0.066*	-0.038	0.088**	0.019	0.121***	-0.053
ZTPI40	-0.004	0.136***	0.086**	0.055	-0.012	0.251***
ZTPI41	0.064*	0.085**	-0.222***	-0.134***	-0.067*	-0.179***
ZTPI42	0.076**	0.035	0.135***	0.040	0.082**	0.010
ZTPI43	-0.034	0.038	0.045	0.012	0.037	0.311***
ZTPI44	-0.065*	0.025	0.210***	0.088**	0.097***	0.071*
ZTPI45	0.040	0.123***	0.057	0.010	0.012	0.200***
ZTPI46	0.149***	0.092**	0.108***	0.080**	0.150***	-0.078**
ZTPI47	-0.069*	-0.050	0.031	0.126***	0.077**	0.030
ZTPI48	0.121***	0.103***	0.004	-0.007	0.021	-0.184***
ZTPI49	0.263***	0.237***	-0.133***	-0.115***	0.001	-0.189***
ZTPI50	-0.103***	-0.016	0.161***	0.349***	0.200***	0.126***
ZTPI51	0.048	0.182***	0.012	-0.016	-0.024	0.112***
ZTPI52	0.036	-0.115***	0.041	-0.073*	0.114***	-0.177***

ZTPI53	0.186***	-0.031	0.110***	0.061*	0.024	-0.107***
ZTPI54	-0.018	-0.008	0.192***	0.423***	0.187***	0.200***
ZTPI55	0.102***	0.256***	0.103***	0.041	0.009	0.051
ZTPI56	0.014	0.070*	-0.155***	-0.076*	-0.081**	-0.066*

Computed correlation used pearson-method with listwise-deletion.

ZTPI7	ZTPI8	ZTPI9	ZTPI10	ZTPI11	ZTPI12	ZTPI13
0.075*	0.098***	-0.082**	0.049	0.091**	0.143***	-0.008
0.145***	-0.004	0.116***	0.181***	0.214***	0.054	0.133***
0.022	0.028	-0.022	0.139***	0.051	0.072*	0.041
-0.021	0.031	0.082**	0.098***	-0.055	0.094**	0.048
0.077**	0.085**	-0.042	-0.031	-0.023	0.016	-0.033
0.064*	-0.169***	0.172***	0.313***	0.043	-0.063*	0.206***
	0.090**	0.013	0.020	0.285***	0.105***	0.019
0.090**		-0.175***	-0.150***	-0.021	0.126***	-0.163***
0.013	-0.175***		0.185***	0.022	-0.088**	0.209***
0.020	-0.150***	0.185***		0.154***	0.040	0.269***
0.285***	-0.021	0.022	0.154***		0.083**	0.121***
0.105***	0.126***	-0.088**	0.040	0.083**		-0.045
0.019	-0.163***	0.209***	0.269***	0.121***	-0.045	
-0.006	0.202***	-0.277***	-0.193***	-0.075*	0.040	-0.147***
0.190***	0.054	0.019	0.091**	0.077**	0.144***	0.113***
-0.096**	0.048	0.011	-0.007	-0.247***	0.053	-0.043
0.079**	0.035	-0.040	0.227***	0.201***	0.080**	0.120***
0.097***	-0.121***	0.165***	0.207***	0.095**	-0.017	0.218***
0.030	0.056	-0.065*	0.052	0.117***	0.135***	0.057
0.259***	-0.009	0.044	0.210***	0.308***	0.071*	0.137***
0.079**	-0.058	0.146***	0.228***	0.120***	-0.016	0.305***
-0.170***	0.001	0.055	0.037	-0.231***	0.045	0.023
-0.022	0.308***	-0.105***	-0.025	-0.045	0.037	-0.076*
0.052	-0.260***	0.220***	0.106***	0.013	-0.118***	0.160***
0.243***	-0.005	0.036	-0.057	0.275***	0.005	0.031
0.072*	0.022	0.104***	0.238***	0.152***	0.078**	0.088**
-0.040	0.034	0.065*	0.060*	-0.130***	0.076**	0.001
0.019	0.122***	-0.165***	-0.043	0.043	0.181***	-0.087**
0.120***	0.060*	-0.035	-0.021	0.045	0.105***	0.004
0.064*	-0.205***	0.188***	0.284***	0.088**	0.013	0.230***
0.024	0.162***	-0.091**	0.074*	0.036	0.180***	-0.072*
0.027	0.114***	-0.099***	0.022	0.119***	0.115***	-0.038
-0.083**	0.090**	-0.040	-0.031	-0.159***	0.001	-0.051
-0.125***	0.005	-0.017	0.047	-0.270***	0.044	-0.015
0.008	0.077**	-0.104***	-0.007	-0.039	0.067*	-0.061*
0.068*	0.073*	-0.008	0.062*	-0.068*	0.075*	0.008
-0.088**	0.193***	-0.146***	-0.163***	-0.035	0.092**	-0.084**
-0.121***	0.107***	-0.135***	-0.149***	-0.050	0.045	-0.060*
-0.068*	0.114***	-0.205***	-0.141***	-0.100***	0.005	-0.067*
0.038	-0.115***	0.141***	0.287***	0.101***	-0.045	0.355***
0.075*	-0.007	0.027	-0.054	0.126***	-0.050	-0.024
0.052	0.170***	-0.113***	0.100***	-0.053	0.136***	-0.067*
0.029	-0.194***	0.134***	0.250***	0.044	-0.091**	0.230***
-0.063*	0.151***	-0.039	0.020	0.053	0.067*	-0.066*
0.017	-0.156***	0.135***	0.206***	0.144***	-0.066*	0.272***
0.044	0.253***	-0.031	-0.008	0.025	0.170***	-0.050
0.113***	0.020	-0.017	-0.074*	-0.040	0.060*	-0.010
-0.033	0.199***	-0.112***	-0.048	-0.036	0.149***	-0.050
0.132***	0.071*	-0.077**	-0.016	0.118***	0.098***	0.055
-0.104***	-0.010	-0.016	0.015	-0.260***	0.030	-0.023
-0.006	-0.078**	0.113***	0.180***	0.056	-0.067*	0.214***
-0.043	0.193***	-0.228***	-0.179***	0.012	0.131***	-0.130***

-0.009	0.103***	-0.154***	-0.119***	-0.044	0.066*	-0.121***
-0.083**	-0.024	0.021	0.047	-0.114***	0.045	0.002
0.042	-0.014	0.095**	0.187***	0.099***	0.092**	0.069*
-0.024	-0.136***	0.120***	0.050	-0.042	-0.072*	0.068*

ZTPI14	ZTPI15	ZTPI16	ZTPI17	ZTPI18	ZTPI19	ZTPI20
0.036	0.084**	0.081**	0.146***	0.106***	0.170***	0.124***
-0.113***	0.194***	0.081**	0.180***	0.206***	0.201***	0.358***
0.175***	0.135***	0.091**	0.032	0.127***	-0.026	0.056
0.101***	0.122***	0.268***	-0.064*	0.092**	-0.037	0.070*
0.146***	0.064*	0.172***	-0.005	0.009	-0.012	0.046
-0.061*	0.129***	0.016	-0.005	0.151***	-0.015	0.039
-0.006	0.190***	-0.096**	0.079**	0.097***	0.030	0.259***
0.202***	0.054	0.048	0.035	-0.121***	0.056	-0.009
-0.277***	0.019	0.011	-0.040	0.165***	-0.065*	0.044
-0.193***	0.091**	-0.007	0.227***	0.207***	0.052	0.210***
-0.075*	0.077**	-0.247***	0.201***	0.095**	0.117***	0.308***
0.040	0.144***	0.053	0.080**	-0.017	0.135***	0.071*
-0.147***	0.113***	-0.043	0.120***	0.218***	0.057	0.137***
	0.039	0.118***	-0.012	-0.157***	-0.034	-0.061*
0.039		0.104***	0.077**	0.114***	-0.002	0.265***
0.118***	0.104***		-0.063*	0.098***	0.009	-0.099***
-0.012	0.077**	-0.063*		0.132***	0.288***	0.318***
-0.157***	0.114***	0.098***	0.132***		0.124***	0.103***
-0.034	-0.002	0.009	0.288***	0.124***		0.169***
-0.061*	0.265***	-0.099***	0.318***	0.103***	0.169***	
-0.123***	0.070*	-0.087**	0.149***	0.291***	0.109***	0.207***
-0.002	0.035	0.344***	-0.029	0.012	0.045	-0.123***
0.136***	0.106***	0.027	0.026	-0.086**	0.015	0.085**
-0.271***	-0.072*	-0.043	-0.089**	0.110***	-0.042	-0.039
-0.140***	-0.026	-0.334***	0.057	-0.030	0.079**	0.132***
-0.125***	0.159***	0.015	0.253***	0.201***	0.131***	0.255***
0.017	0.116***	0.249***	-0.001	0.066*	0.016	-0.016
0.197***	0.031	0.068*	0.080**	-0.090**	0.075*	0.031
0.083**	0.216***	0.138***	0.028	0.049	0.078**	0.150***
-0.187***	0.164***	0.044	0.074*	0.229***	0.022	0.142***
0.051	0.087**	0.036	0.178***	-0.013	0.123***	0.105***
0.106***	0.069*	-0.087**	0.215***	-0.060*	0.103***	0.150***
0.125***	0.119***	0.223***	-0.053	-0.028	-0.065*	-0.084**
0.102***	0.081**	0.453***	-0.078**	0.019	-0.064*	-0.112***
0.227***	0.076*	0.113***	-0.083**	-0.096**	-0.041	0.019
0.150***	0.165***	0.230***	-0.013	0.060*	-0.048	0.006
0.279***	0.028	0.154***	0.018	0.013	0.111***	0.006
0.289***	0.110***	0.154***	-0.026	-0.116***	-0.018	-0.043
0.323***	0.015	0.152***	-0.019	-0.098***	-0.026	-0.006
-0.122***	0.155***	0.001	0.110***	0.232***	0.064*	0.207***
-0.155***	0.055	-0.186***	0.010	-0.042	0.102***	0.088**
0.124***	0.068*	0.059*	0.156***	-0.062*	0.085**	0.038
-0.192***	0.067*	0.052	0.049	0.154***	-0.008	0.085**
0.123***	0.204***	0.044	0.082**	-0.013	0.052	0.100***
-0.082**	0.074*	0.052	0.099***	0.194***	0.068*	0.148***
0.114***	0.152***	0.089**	0.086**	0.053	0.059*	0.105***
0.140***	0.216***	0.124***	-0.105***	-0.028	-0.035	-0.015
0.164***	0.071*	0.091**	0.153***	-0.012	0.070*	0.103***
-0.033	0.104***	0.014	0.173***	0.092**	0.238***	0.164***
0.135***	0.134***	0.486***	-0.117***	-0.013	-0.125***	-0.108***
-0.116***	0.083**	0.014	0.094**	0.194***	0.099***	0.121***
0.223***	-0.026	-0.035	-0.013	-0.138***	-0.071*	-0.093**

0.303***	-0.012	0.095**	-0.017	-0.119***	-0.049	-0.039
0.172***	0.135***	0.292***	-0.115***	0.042	-0.034	-0.047
-0.063*	0.155***	0.051	0.169***	0.196***	0.123***	0.239***
-0.268***	-0.117***	-0.033	-0.049	0.097***	0.062*	-0.032

ZTPI21	ZTPI22	ZTPI23	ZTPI24	ZTPI25	ZTPI26	ZTPI27
0.015	-0.086**	-0.013	-0.029	0.083**	0.198***	-0.082**
0.125***	-0.024	0.026	0.077**	0.115***	0.247***	-0.011
0.076**	0.059*	0.172***	-0.111***	-0.190***	0.105***	0.170***
0.015	0.151***	0.026	-0.025	-0.231***	0.043	0.346***
-0.022	0.032	0.030	-0.082**	-0.111***	-0.024	0.105***
0.164***	0.076**	-0.009	0.267***	-0.098***	0.027	0.176***
0.079**	-0.170***	-0.022	0.052	0.243***	0.072*	-0.040
-0.058	0.001	0.308***	-0.260***	-0.005	0.022	0.034
0.146***	0.055	-0.105***	0.220***	0.036	0.104***	0.065*
0.228***	0.037	-0.025	0.106***	-0.057	0.238***	0.060*
0.120***	-0.231***	-0.045	0.013	0.275***	0.152***	-0.130***
-0.016	0.045	0.037	-0.118***	0.005	0.078**	0.076**
0.305***	0.023	-0.076*	0.160***	0.031	0.088**	0.001
-0.123***	-0.002	0.136***	-0.271***	-0.140***	-0.125***	0.017
0.070*	0.035	0.106***	-0.072*	-0.026	0.159***	0.116***
-0.087**	0.344***	0.027	-0.043	-0.334***	0.015	0.249***
0.149***	-0.029	0.026	-0.089**	0.057	0.253***	-0.001
0.291***	0.012	-0.086**	0.110***	-0.030	0.201***	0.066*
0.109***	0.045	0.015	-0.042	0.079**	0.131***	0.016
0.207***	-0.123***	0.085**	-0.039	0.132***	0.255***	-0.016
	-0.003	-0.075*	0.081**	0.071*	0.147***	0.017
-0.003		0.095**	-0.077**	-0.388***	0.017	0.268***
-0.075*	0.095**		-0.248***	-0.092**	0.070*	0.147***
0.081**	-0.077**	-0.248***		0.169***	-0.078**	-0.032
0.071*	-0.388***	-0.092**	0.169***		0.013	-0.289***
0.147***	0.017	0.070*	-0.078**	0.013		0.037
0.017	0.268***	0.147***	-0.032	-0.289***	0.037	
-0.077**	0.029	0.119***	-0.179***	-0.074*	0.050	0.037
0.019	0.071*	0.073*	-0.066*	-0.089**	0.102***	0.114***
0.211***	0.037	-0.157***	0.151***	-0.030	0.195***	0.088**
-0.039	0.115***	0.167***	-0.132***	-0.041	0.210***	0.085**
0.056	0.018	0.134***	-0.249***	0.030	0.152***	-0.011
-0.076**	0.242***	0.174***	-0.170***	-0.250***	-0.004	0.190***
-0.036	0.395***	0.092**	-0.145***	-0.424***	0.034	0.303***
-0.064*	0.072*	0.233***	-0.164***	-0.177***	-0.011	0.174***
0.048	0.177***	0.137***	-0.081**	-0.211***	0.083**	0.300***
-0.013	0.020	0.051	-0.247***	-0.097***	0.030	0.019
-0.090**	0.127***	0.182***	-0.177***	-0.181***	-0.105***	0.152***
-0.113***	0.060*	0.139***	-0.192***	-0.187***	-0.113***	0.043
0.338***	-0.018	-0.063*	0.128***	-0.044	0.134***	0.052
0.010	-0.152***	-0.146***	0.058*	0.239***	0.027	-0.150***
-0.099***	0.109***	0.224***	-0.142***	-0.082**	0.063*	0.066*
0.101***	0.034	-0.080**	0.248***	-0.016	0.102***	0.084**
0.048	0.082**	0.284***	-0.201***	-0.160***	0.090**	0.133***
0.205***	0.076**	-0.033	0.138***	-0.067*	0.133***	0.059*
0.002	0.056	0.200***	-0.195***	-0.003	0.159***	0.134***
-0.019	0.121***	0.028	-0.108***	-0.156***	-0.103***	0.146***
-0.004	0.015	0.266***	-0.233***	-0.030	0.140***	0.054
0.124***	-0.077**	-0.077**	-0.021	0.182***	0.085**	-0.150***
-0.079**	0.377***	0.124***	-0.106***	-0.420***	-0.018	0.358***
0.237***	0.077**	-0.046	0.094**	0.011	0.158***	0.018
-0.106***	-0.002	0.175***	-0.224***	-0.001	-0.094**	-0.071*

-0.136***	-0.002	0.067*	-0.180***	-0.135***	0.027	-0.009
-0.052	0.235***	0.104***	-0.068*	-0.310***	0.015	0.397***
0.187***	0.027	0.126***	0.033	0.022	0.330***	0.040
0.044	-0.084**	-0.200***	0.159***	0.092**	-0.032	-0.077**

ZTPI28	ZTPI29	ZTPI30	ZTPI31	ZTPI32	ZTPI33	ZTPI34
0.143***	0.114***	0.041	0.107***	0.044	-0.070*	-0.036
0.059*	0.213***	0.094**	0.032	0.052	-0.054	-0.058*
0.047	0.077**	0.052	0.101***	0.084**	0.151***	0.189***
0.025	0.073*	0.032	0.028	-0.019	0.197***	0.270***
0.057	0.046	-0.010	-0.038	-0.014	0.133***	0.107***
-0.072*	-0.050	0.254***	-0.006	-0.133***	0.008	0.106***
0.019	0.120***	0.064*	0.024	0.027	-0.083**	-0.125***
0.122***	0.060*	-0.205***	0.162***	0.114***	0.090**	0.005
-0.165***	-0.035	0.188***	-0.091**	-0.099***	-0.040	-0.017
-0.043	-0.021	0.284***	0.074*	0.022	-0.031	0.047
0.043	0.045	0.088**	0.036	0.119***	-0.159***	-0.270***
0.181***	0.105***	0.013	0.180***	0.115***	0.001	0.044
-0.087**	0.004	0.230***	-0.072*	-0.038	-0.051	-0.015
0.197***	0.083**	-0.187***	0.051	0.106***	0.125***	0.102***
0.031	0.216***	0.164***	0.087**	0.069*	0.119***	0.081**
0.068*	0.138***	0.044	0.036	-0.087**	0.223***	0.453***
0.080**	0.028	0.074*	0.178***	0.215***	-0.053	-0.078**
-0.090**	0.049	0.229***	-0.013	-0.060*	-0.028	0.019
0.075*	0.078**	0.022	0.123***	0.103***	-0.065*	-0.064*
0.031	0.150***	0.142***	0.105***	0.150***	-0.084**	-0.112***
-0.077**	0.019	0.211***	-0.039	0.056	-0.076**	-0.036
0.029	0.071*	0.037	0.115***	0.018	0.242***	0.395***
0.119***	0.073*	-0.157***	0.167***	0.134***	0.174***	0.092**
-0.179***	-0.066*	0.151***	-0.132***	-0.249***	-0.170***	-0.145***
-0.074*	-0.089**	-0.030	-0.041	0.030	-0.250***	-0.424***
0.050	0.102***	0.195***	0.210***	0.152***	-0.004	0.034
0.037	0.114***	0.088**	0.085**	-0.011	0.190***	0.303***
	0.099***	-0.089**	0.118***	0.214***	0.100***	0.026
0.099***		0.106***	-0.017	0.040	0.053	0.073*
-0.089**	0.106***		0.024	-0.067*	0.027	0.106***
0.118***	-0.017	0.024		0.218***	0.030	0.075*
0.214***	0.040	-0.067*	0.218***		0.079**	-0.056
0.100***	0.053	0.027	0.030	0.079**		0.293***
0.026	0.073*	0.106***	0.075*	-0.056	0.293***	
0.173***	0.080**	-0.019	0.082**	0.063*	0.211***	0.224***
0.075*	0.139***	0.125***	0.104***	0.021	0.182***	0.363***
0.211***	0.098***	-0.085**	0.074*	0.156***	0.188***	0.092**
0.140***	0.165***	-0.004	0.046	0.142***	0.174***	0.129***
0.155***	0.073*	-0.131***	0.044	0.113***	0.148***	0.020
-0.101***	-0.006	0.276***	0.025	-0.004	-0.005	-0.034
-0.123***	0.000	-0.052	-0.046	-0.041	-0.166***	-0.224***
0.126***	-0.012	-0.053	0.489***	0.173***	0.087**	0.080**
-0.098***	0.073*	0.208***	-0.054	-0.102***	0.010	0.072*
0.105***	0.052	-0.048	0.143***	0.126***	0.155***	0.095**
-0.098***	-0.019	0.263***	0.017	-0.001	0.005	0.030
0.166***	0.117***	-0.047	0.120***	0.124***	0.157***	0.049
0.074*	0.142***	-0.007	-0.028	-0.012	0.183***	0.072*
0.141***	0.076*	-0.118***	0.187***	0.284***	0.119***	0.020
0.072*	0.146***	-0.029	0.001	0.117***	-0.016	-0.093**
0.032	0.141***	0.044	0.049	-0.011	0.201***	0.513***
-0.082**	0.020	0.153***	0.090**	0.024	0.002	0.017
0.130***	0.071*	-0.191***	0.078**	0.120***	0.051	-0.014

0.220***	0.076**	-0.044	0.072*	0.020	0.156***	0.146***
0.058*	0.125***	0.092**	0.021	0.026	0.248***	0.363***
-0.003	0.110***	0.192***	0.161***	0.093**	0.002	0.049
-0.211***	-0.074*	-0.039	-0.115***	-0.118***	-0.069*	-0.098***

ZTPI35	ZTPI36	ZTPI37	ZTPI38	ZTPI39	ZTPI40	ZTPI41
-0.008	-0.098***	0.142***	0.007	0.066*	-0.004	0.064*
-0.010	-0.047	0.050	0.005	-0.038	0.136***	0.085**
0.265***	0.194***	0.024	0.191***	0.088**	0.086**	-0.222***
0.164***	0.277***	0.094**	0.088**	0.019	0.055	-0.134***
0.149***	0.126***	0.063*	0.156***	0.121***	-0.012	-0.067*
0.089**	0.197***	-0.225***	-0.035	-0.053	0.251***	-0.179***
0.008	0.068*	-0.088**	-0.121***	-0.068*	0.038	0.075*
0.077**	0.073*	0.193***	0.107***	0.114***	-0.115***	-0.007
-0.104***	-0.008	-0.146***	-0.135***	-0.205***	0.141***	0.027
-0.007	0.062*	-0.163***	-0.149***	-0.141***	0.287***	-0.054
-0.039	-0.068*	-0.035	-0.050	-0.100***	0.101***	0.126***
0.067*	0.075*	0.092**	0.045	0.005	-0.045	-0.050
-0.061*	0.008	-0.084**	-0.060*	-0.067*	0.355***	-0.024
0.227***	0.150***	0.279***	0.289***	0.323***	-0.122***	-0.155***
0.076*	0.165***	0.028	0.110***	0.015	0.155***	0.055
0.113***	0.230***	0.154***	0.154***	0.152***	0.001	-0.186***
-0.083**	-0.013	0.018	-0.026	-0.019	0.110***	0.010
-0.096**	0.060*	0.013	-0.116***	-0.098***	0.232***	-0.042
-0.041	-0.048	0.111***	-0.018	-0.026	0.064*	0.102***
0.019	0.006	0.006	-0.043	-0.006	0.207***	0.088**
-0.064*	0.048	-0.013	-0.090**	-0.113***	0.338***	0.010
0.072*	0.177***	0.020	0.127***	0.060*	-0.018	-0.152***
0.233***	0.137***	0.051	0.182***	0.139***	-0.063*	-0.146***
-0.164***	-0.081**	-0.247***	-0.177***	-0.192***	0.128***	0.058*
-0.177***	-0.211***	-0.097***	-0.181***	-0.187***	-0.044	0.239***
-0.011	0.083**	0.030	-0.105***	-0.113***	0.134***	0.027
0.174***	0.300***	0.019	0.152***	0.043	0.052	-0.150***
0.173***	0.075*	0.211***	0.140***	0.155***	-0.101***	-0.123***
0.080**	0.139***	0.098***	0.165***	0.073*	-0.006	0.000
-0.019	0.125***	-0.085**	-0.004	-0.131***	0.276***	-0.052
0.082**	0.104***	0.074*	0.046	0.044	0.025	-0.046
0.063*	0.021	0.156***	0.142***	0.113***	-0.004	-0.041
0.211***	0.182***	0.188***	0.174***	0.148***	-0.005	-0.166***
0.224***	0.363***	0.092**	0.129***	0.020	-0.034	-0.224***
	0.227***	0.158***	0.151***	0.151***	0.035	-0.235***
0.227***		0.136***	0.140***	0.075*	0.042	-0.186***
0.158***	0.136***		0.237***	0.253***	-0.080**	-0.049
0.151***	0.140***	0.237***		0.418***	-0.022	-0.115***
0.151***	0.075*	0.253***	0.418***		-0.044	-0.154***
0.035	0.042	-0.080**	-0.022	-0.044		-0.006
-0.235***	-0.186***	-0.049	-0.115***	-0.154***	-0.006	
0.140***	0.078**	-0.007	0.117***	0.121***	-0.022	-0.102***
-0.082**	0.058*	-0.194***	-0.085**	-0.114***	0.225***	-0.006
0.175***	0.130***	0.037	0.128***	0.087**	0.019	-0.101***
-0.003	0.032	-0.001	-0.019	-0.027	0.367***	-0.038
0.111***	0.089**	0.153***	0.141***	0.085**	-0.025	-0.077**
0.159***	0.137***	0.126***	0.167***	0.118***	-0.004	-0.003
0.120***	-0.008	0.202***	0.108***	0.104***	-0.047	-0.032
-0.147***	-0.082**	0.166***	0.060*	0.029	0.018	0.180***
0.215***	0.413***	0.051	0.179***	0.170***	0.035	-0.173***
-0.088**	0.011	0.005	-0.066*	-0.095**	0.304***	0.025
0.122***	-0.012	0.186***	0.167***	0.140***	-0.211***	-0.023

0.210***	0.084**	0.233***	0.210***	0.187***	-0.103***	-0.086**
0.245***	0.371***	0.053	0.197***	0.113***	0.010	-0.188***
0.022	0.064*	0.065*	-0.031	-0.133***	0.118***	-0.012
-0.272***	-0.103***	-0.078**	-0.158***	-0.188***	0.059*	0.157***

ZTPI42	ZTPI43	ZTPI44	ZTPI45	ZTPI46	ZTPI47	ZTPI48
0.076**	-0.034	-0.065*	0.040	0.149***	-0.069*	0.121***
0.035	0.038	0.025	0.123***	0.092**	-0.050	0.103***
0.135***	0.045	0.210***	0.057	0.108***	0.031	0.004
0.040	0.012	0.088**	0.010	0.080**	0.126***	-0.007
0.082**	0.037	0.097***	0.012	0.150***	0.077**	0.021
0.010	0.311***	0.071*	0.200***	-0.078**	0.030	-0.184***
0.052	0.029	-0.063*	0.017	0.044	0.113***	-0.033
0.170***	-0.194***	0.151***	-0.156***	0.253***	0.020	0.199***
-0.113***	0.134***	-0.039	0.135***	-0.031	-0.017	-0.112***
0.100***	0.250***	0.020	0.206***	-0.008	-0.074*	-0.048
-0.053	0.044	0.053	0.144***	0.025	-0.040	-0.036
0.136***	-0.091**	0.067*	-0.066*	0.170***	0.060*	0.149***
-0.067*	0.230***	-0.066*	0.272***	-0.050	-0.010	-0.050
0.124***	-0.192***	0.123***	-0.082**	0.114***	0.140***	0.164***
0.068*	0.067*	0.204***	0.074*	0.152***	0.216***	0.071*
0.059*	0.052	0.044	0.052	0.089**	0.124***	0.091**
0.156***	0.049	0.082**	0.099***	0.086**	-0.105***	0.153***
-0.062*	0.154***	-0.013	0.194***	0.053	-0.028	-0.012
0.085**	-0.008	0.052	0.068*	0.059*	-0.035	0.070*
0.038	0.085**	0.100***	0.148***	0.105***	-0.015	0.103***
-0.099***	0.101***	0.048	0.205***	0.002	-0.019	-0.004
0.109***	0.034	0.082**	0.076**	0.056	0.121***	0.015
0.224***	-0.080**	0.284***	-0.033	0.200***	0.028	0.266***
-0.142***	0.248***	-0.201***	0.138***	-0.195***	-0.108***	-0.233***
-0.082**	-0.016	-0.160***	-0.067*	-0.003	-0.156***	-0.030
0.063*	0.102***	0.090**	0.133***	0.159***	-0.103***	0.140***
0.066*	0.084**	0.133***	0.059*	0.134***	0.146***	0.054
0.126***	-0.098***	0.105***	-0.098***	0.166***	0.074*	0.141***
-0.012	0.073*	0.052	-0.019	0.117***	0.142***	0.076*
-0.053	0.208***	-0.048	0.263***	-0.047	-0.007	-0.118***
0.489***	-0.054	0.143***	0.017	0.120***	-0.028	0.187***
0.173***	-0.102***	0.126***	-0.001	0.124***	-0.012	0.284***
0.087**	0.010	0.155***	0.005	0.157***	0.183***	0.119***
0.080**	0.072*	0.095**	0.030	0.049	0.072*	0.020
0.140***	-0.082**	0.175***	-0.003	0.111***	0.159***	0.120***
0.078**	0.058*	0.130***	0.032	0.089**	0.137***	-0.008
-0.007	-0.194***	0.037	-0.001	0.153***	0.126***	0.202***
0.117***	-0.085**	0.128***	-0.019	0.141***	0.167***	0.108***
0.121***	-0.114***	0.087**	-0.027	0.085**	0.118***	0.104***
-0.022	0.225***	0.019	0.367***	-0.025	-0.004	-0.047
-0.102***	-0.006	-0.101***	-0.038	-0.077**	-0.003	-0.032
	-0.016	0.199***	-0.044	0.125***	-0.035	0.150***
-0.016		0.091**	0.215***	-0.026	-0.028	-0.090**
0.199***	0.091**		-0.003	0.191***	0.062*	0.130***
-0.044	0.215***	-0.003		-0.027	0.013	-0.040
0.125***	-0.026	0.191***	-0.027		0.125***	0.244***
-0.035	-0.028	0.062*	0.013	0.125***		0.054
0.150***	-0.090**	0.130***	-0.040	0.244***	0.054	
-0.098***	-0.057	-0.122***	-0.032	0.062*	0.052	0.060*
0.113***	0.080**	0.160***	0.010	0.059*	0.164***	0.008
0.033	0.185***	-0.028	0.302***	-0.083**	-0.060*	-0.017
0.138***	-0.153***	0.118***	-0.148***	0.163***	0.042	0.157***

0.044	-0.139***	0.046	-0.031	0.166***	0.092**	0.097***
0.115***	0.032	0.134***	-0.005	0.113***	0.212***	0.064*
0.142***	0.130***	0.216***	0.107***	0.123***	-0.041	0.169***
-0.152***	0.064*	-0.218***	0.039	-0.102***	-0.073*	-0.136***

ZTPI49	ZTPI50	ZTPI51	ZTPI52	ZTPI53	ZTPI54	ZTPI55
0.263***	-0.103***	0.048	0.036	0.186***	-0.018	0.102***
0.237***	-0.016	0.182***	-0.115***	-0.031	-0.008	0.256***
-0.133***	0.161***	0.012	0.041	0.110***	0.192***	0.103***
-0.115***	0.349***	-0.016	-0.073*	0.061*	0.423***	0.041
0.001	0.200***	-0.024	0.114***	0.024	0.187***	0.009
-0.189***	0.126***	0.112***	-0.177***	-0.107***	0.200***	0.051
0.132***	-0.104***	-0.006	-0.043	-0.009	-0.083**	0.042
0.071*	-0.010	-0.078**	0.193***	0.103***	-0.024	-0.014
-0.077**	-0.016	0.113***	-0.228***	-0.154***	0.021	0.095**
-0.016	0.015	0.180***	-0.179***	-0.119***	0.047	0.187***
0.118***	-0.260***	0.056	0.012	-0.044	-0.114***	0.099***
0.098***	0.030	-0.067*	0.131***	0.066*	0.045	0.092**
0.055	-0.023	0.214***	-0.130***	-0.121***	0.002	0.069*
-0.033	0.135***	-0.116***	0.223***	0.303***	0.172***	-0.063*
0.104***	0.134***	0.083**	-0.026	-0.012	0.135***	0.155***
0.014	0.486***	0.014	-0.035	0.095**	0.292***	0.051
0.173***	-0.117***	0.094**	-0.013	-0.017	-0.115***	0.169***
0.092**	-0.013	0.194***	-0.138***	-0.119***	0.042	0.196***
0.238***	-0.125***	0.099***	-0.071*	-0.049	-0.034	0.123***
0.164***	-0.108***	0.121***	-0.093**	-0.039	-0.047	0.239***
0.124***	-0.079**	0.237***	-0.106***	-0.136***	-0.052	0.187***
-0.077**	0.377***	0.077**	-0.002	-0.002	0.235***	0.027
-0.077**	0.124***	-0.046	0.175***	0.067*	0.104***	0.126***
-0.021	-0.106***	0.094**	-0.224***	-0.180***	-0.068*	0.033
0.182***	-0.420***	0.011	-0.001	-0.135***	-0.310***	0.022
0.085**	-0.018	0.158***	-0.094**	0.027	0.015	0.330***
-0.150***	0.358***	0.018	-0.071*	-0.009	0.397***	0.040
0.072*	0.032	-0.082**	0.130***	0.220***	0.058*	-0.003
0.146***	0.141***	0.020	0.071*	0.076**	0.125***	0.110***
-0.029	0.044	0.153***	-0.191***	-0.044	0.092**	0.192***
0.001	0.049	0.090**	0.078**	0.072*	0.021	0.161***
0.117***	-0.011	0.024	0.120***	0.020	0.026	0.093**
-0.016	0.201***	0.002	0.051	0.156***	0.248***	0.002
-0.093**	0.513***	0.017	-0.014	0.146***	0.363***	0.049
-0.147***	0.215***	-0.088**	0.122***	0.210***	0.245***	0.022
-0.082**	0.413***	0.011	-0.012	0.084**	0.371***	0.064*
0.166***	0.051	0.005	0.186***	0.233***	0.053	0.065*
0.060*	0.179***	-0.066*	0.167***	0.210***	0.197***	-0.031
0.029	0.170***	-0.095**	0.140***	0.187***	0.113***	-0.133***
0.018	0.035	0.304***	-0.211***	-0.103***	0.010	0.118***
0.180***	-0.173***	0.025	-0.023	-0.086**	-0.188***	-0.012
-0.098***	0.113***	0.033	0.138***	0.044	0.115***	0.142***
-0.057	0.080**	0.185***	-0.153***	-0.139***	0.032	0.130***
-0.122***	0.160***	-0.028	0.118***	0.046	0.134***	0.216***
-0.032	0.010	0.302***	-0.148***	-0.031	-0.005	0.107***
0.062*	0.059*	-0.083**	0.163***	0.166***	0.113***	0.123***
0.052	0.164***	-0.060*	0.042	0.092**	0.212***	-0.041
0.060*	0.008	-0.017	0.157***	0.097***	0.064*	0.169***
	-0.118***	0.048	0.025	0.070*	-0.136***	0.110***
-0.118***		0.028	0.001	0.102***	0.456***	0.034
0.048	0.028		-0.114***	-0.104***	-0.038	0.156***
0.025	0.001	-0.114***		0.202***	-0.033	-0.057

0.070*	0.102***	-0.104***	0.202***		0.153***	0.001
-0.136***	0.456***	-0.038	-0.033	0.153***		0.059*
0.110***	0.034	0.156***	-0.057	0.001	0.059*	
0.154***	-0.111***	0.136***	-0.163***	-0.125***	-0.153***	-0.103***

ZTPI56
0.014
0.070*
-0.155***
-0.076*
-0.081**
-0.066*
-0.024
-0.136***
0.120***
0.050
-0.042
-0.072*
0.068*
-0.268***
-0.117***
-0.033
-0.049
0.097***
0.062*
-0.032
0.044
-0.084**
-0.200***
0.159***
0.092**
-0.032
-0.077**
-0.211***
-0.074*
-0.039
-0.115***
-0.118***
-0.069*
-0.098***
-0.272***
-0.103***
-0.078**
-0.158***
-0.188***
0.059*
0.157***
-0.152***
0.064*
-0.218***
0.039
-0.102***
-0.073*
-0.136***
0.154***
-0.111***
0.136***
-0.163***

-0.125***
-0.153***
-0.103***
