# Supplementary material for: What Is the Structure of Time? A Study on Time Perspective in the United States, Poland, and Nigeria
Source: Front Psychol. 2018 Nov 1;9:2078. doi: 10.3389/fpsyg.2018.02078 (PMC6221929; doi:10.3389/fpsyg.2018.02078)
Supplement: Supplementary file 2 [file Table_1.doc]

Appendix I

ESEM factor loadings for ZTPI items in the USA, Poland and Nigeria sample
